# CSF Biomarkers Reflecting Protein Pathology and Axonal Degeneration Are Associated with Memory, Attentional, and Executive Functioning in Early-Stage Parkinson′s Disease

**DOI:** 10.3390/ijms21228519

**Published:** 2020-11-12

**Authors:** Linda P. Oosterveld, Tessa I. Kuiper, Nour K. Majbour, Inge M. W. Verberk, Karin D. van Dijk, Jos W. R. Twisk, Omar M. El-Agnaf, Charlotte E. Teunissen, Henry C. Weinstein, Martin Klein, Henk W. Berendse, Wilma D. J. van de Berg

**Affiliations:** 1 Department of Anatomy and Neurosciences, Amsterdam Neuroscience, Amsterdam UMC, Vrije Universiteit Amsterdam, De Boelelaan 1117, 1081 HV Amsterdam, The Netherlands; l.oosterveld@amsterdamumc.nl (L.P.O.); tessa-kuiper@hotmail.com (T.I.K.); 2 Neurological Disorders Research Center, Qatar Biomedical Research Institute (QBRI), Hamad Bin Khalifa University (HBKU), Qatar Foundation, PO Box 5825 Doha, Qatar; nmajbour@hbku.edu.qa (N.K.M.); oelagnaf@hbku.edu.qa (O.M.E.-A.); 3 Neurochemistry Lab, Department of Clinical Chemistry, Amsterdam Neuroscience, Amsterdam UMC, Vrije Universiteit Amsterdam, De Boelelaan 1117, 1081 HV Amsterdam, The Netherlands; i.verberk@amsterdamumc.nl (I.M.W.V.); c.teunissen@amsterdamumc.nl (C.E.T.); 4 Department of Neurology, Amsterdam Neuroscience, Amsterdam UMC, Vrije Universiteit Amsterdam, De Boelelaan 1117, 1081 HV Amsterdam, The Netherlands; kvdijk@sein.nl (K.D.v.D.); h.weinstein@olvg.nl (H.C.W.); h.berendse@amsterdamumc.nl (H.W.B.); 5 Department of Epidemiology and Biostatistics, Amsterdam Neuroscience, Amsterdam UMC, Vrije Universiteit Amsterdam, De Boelelaan 1117, 1081 HV Amsterdam, The Netherlands; jwr.twisk@amsterdamumc.nl; 6 Department of Neurology, OLVG, West, Jan Tooropstraat 164, 1061 AE Amsterdam, The Netherlands; 7 Department of Medical Psychology, Amsterdam Neuroscience, Amsterdam UMC, Vrije Universiteit Amsterdam, De Boelelaan 1117, 1081 HV Amsterdam, The Netherlands; m.klein@amsterdamumc.nl

**Keywords:** CSF biomarkers, cognitive impairment, neuropsychological testing, Parkinson′s disease

## Abstract

In early-stage Parkinson′s disease (PD), cognitive impairment is common, and a variety of cognitive domains including memory, attention, and executive functioning may be affected. Cerebrospinal fluid (CSF) biomarkers are potential markers of cognitive functioning. We aimed to explore whether CSF α-synuclein species, neurofilament light chain, amyloid-β_42_, and tau are associated with cognitive performance in early-stage PD patients. CSF levels of total-α-synuclein and phosphorylated-α-synuclein, neurofilament light chain, amyloid-β_42_, and total-tau and phosphorylated-tau were measured in 26 PD patients (disease duration ≤5 years and Hoehn and Yahr stage 1–2.5). Multivariable linear regression models, adjusted for age, gender, and educational level, were used to assess the relationship between CSF biomarker levels and memory, attention, executive and visuospatial function, and language performance scores. In 26 early-stage PD patients, attention and memory were the most commonly affected domains. A higher CSF phosphorylated-α-synuclein/total-α-synuclein ratio was associated with better executive functioning (sβ = 0.40). Higher CSF neurofilament light was associated with worse memory (sβ = −0.59), attentional (sβ = −0.32), and executive functioning (sβ = −0.35). Reduced CSF amyloid-β_42_ levels were associated with poorer attentional functioning (sβ = 0.35). Higher CSF phosphorylated-tau was associated with worse language functioning (sβ = −0.33). Thus, CSF biomarker levels, in particular neurofilament light, were related to the most commonly affected cognitive domains in early-stage PD. This indicates that CSF biomarker levels may identify early-stage PD patients who are at an increased risk of developing cognitive impairment.

## 1. Introduction

Cognitive impairment is a common non-motor symptom in early-stage Parkinson′s disease (PD), and can have a major negative impact on the patient′s quality of life [1]. At the time of diagnosis, about 20–35% of PD patients meet criteria for mild cognitive impairment (PD-MCI) [2], which is associated with an increased risk of developing comorbid dementia [3]. However, the timing of onset as well as the nature of cognitive dysfunction vary widely among PD patients [3]. At early stages of disease, deficits in processing speed, attention, executive functioning, and working memory seem to predominate, while, in PD patients with dementia, other functions such as visuospatial perception and memory are more profoundly affected [4]. Nevertheless, even in early-stage PD, there is variability in the affected cognitive domains, as some studies demonstrated memory, not executive functioning, to be among the most commonly affected domains [5,6,7,8,9].

To identify patients who are at an increased risk to develop cognitive impairment, sensitive and specific biofluid biomarkers that reflect underlying pathophysiological mechanisms of cognitive dysfunction are needed. A variety of mechanisms are proposed to contribute to cognitive dysfunction in PD, such as misfolding of the proteins α-synuclein, amyloid-β, and tau, axonal degeneration, synaptic dysfunction, neuro-inflammation, and abnormalities of brain connectivity [10]. In search for cerebrospinal fluid (CSF) biomarkers of cognitive impairment, CSF total α-synuclein (t-α-syn), amyloid-β_42_ (Aβ42), total tau (t-tau), and tau phosphorylated at threonine 181 (p-tau) have been extensively studied in different cohorts [7,10,11,12]. However, the majority of previous cross-sectional studies analyzed CSF biomarkers in PD populations with a long disease duration, while it is important to study these relations in the earliest stages of disease to identify patients who are at an increased risk for developing cognitive impairment. From the few studies that investigated cognitive impairment in early-stage PD patients, the most consistent finding was a positive association between CSF Aβ42 levels and memory functioning [7,11,12]. However, the association between CSF t-α-syn, t-tau, or p-tau and cognitive impairment is less clear [7,11,13]. In one de novo PD cohort, reduced CSF t-α-syn was associated with worse performance in the executive-attention domain [13]. Yet, similar studies in early-stage PD did not find a relation between CSF t-α-syn and cognitive performance [7,11]. Likewise, most early-stage PD studies reported no associations between CSF t-tau or p-tau levels and cognitive impairment [7,11,12,13]. CSF levels of α-syn phosphorylated at residue Ser 129 (p-α-syn), which is a post-translationally modified form of α-syn, is considered as a marker reflecting the ongoing disease process and might be better related to cognitive impairment than CSF t-α-syn [14]. CSF neurofilament light chain (NfL), which is a marker of axonal injury, is increased in a broad spectrum of neurological disorders and also in Alzheimer′s disease (AD), frontotemporal dementia, and vascular dementia [15]. In PD, elevated CSF NfL seems to be associated with future PD dementia [16]. Since axonal degeneration is an early-stage feature of PD, CSF NfL might also serve as a marker of cognitive functioning in the early stages of PD [17]. However, whether CSF p-α-syn and CSF NfL are associated with cognitive functioning in early PD remains to be determined.

The aim of this study was to explore whether CSF levels of t-α-syn, p-α-syn, p-α-syn/t-α-syn, NfL, Aβ42, t-tau, and p-tau are associated with cognitive performance in early-stage PD patients. First, we determined which of the five cognitive domains, involving memory, attention, executive functioning, visuospatial functions, and language, were most commonly impaired in an early-stage PD cohort. Next, we investigated whether CSF t-α-syn, p-α-syn, p-α-syn/t-α-syn, NfL, Aβ42, t-tau, and p-tau were associated with cognitive functioning of the previously mentioned domains in our cohort.

## 2. Results

### 2.1. Cognitive Functioning in Early-Stage PD

Twenty-six early-stage PD patients with neuropsychological test scores and CSF biomarker levels were included in the study. Table 1 presents the demographical data, clinical characteristics, and CSF biomarker levels. Mean disease duration was 2.3 years. Regarding (motor) disease severity, mean Unified Parkinson′s Disease Rating Scale Part III (UPDRS-III) score was 22% and 50% of patients who were in Hoehn & Yahr (HY) stage 2. With a mean Mini-Mental State Examination (MMSE) score of 29 out of 30, global cognitive performance of the cohort was good. Table 2 presents neuropsychological tests scores and z-scores of the five cognitive domains.

PD-MCI criteria were met by 3 out of 26 patients (11.5%) [2] and one patient scored at the level of PD dementia (3.8%). The attentional domain was most commonly affected (*n* = 10, 38.5%), followed by memory functioning (*n* = 6, 23.1%). Performance on executive function tests was relatively good with impairment in only one patient (3.8%). The frequency of impairment of visuospatial functions was 7.7% (*n* = 2). None of the patients had language difficulties.

### 2.2. Associations of CSF Biomarker Levels with Cognitive Performance

**Figure 1:**
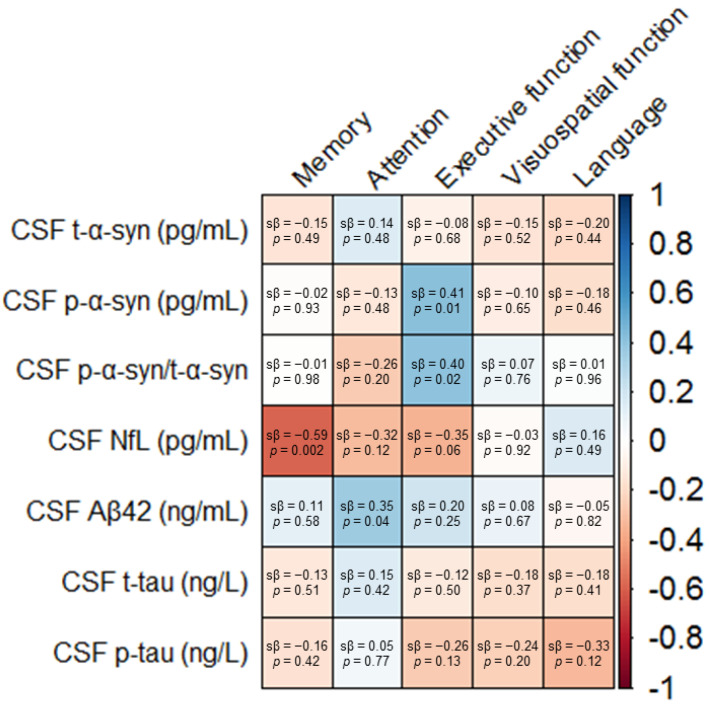
Associations of the cerebrospinal (CSF) biomarker levels with cognitive domain scores in early-stage Parkinson′s disease patients. Data are standardized β coefficients from linear regression models, adjusted for age, gender, and level of education. Color intensity is based on the standardized β coefficients. Abbreviations: CSF, cerebrospinal fluid. t-α-syn, total α-synuclein. p-α-syn, phosphorylated α-synuclein. NfL, neurofilament light chain. Aβ42, amyloid-β42. t-tau, total tau. p-tau, phosphorylated tau. sβ, standardized β-coefficient.

**Figure 2:**
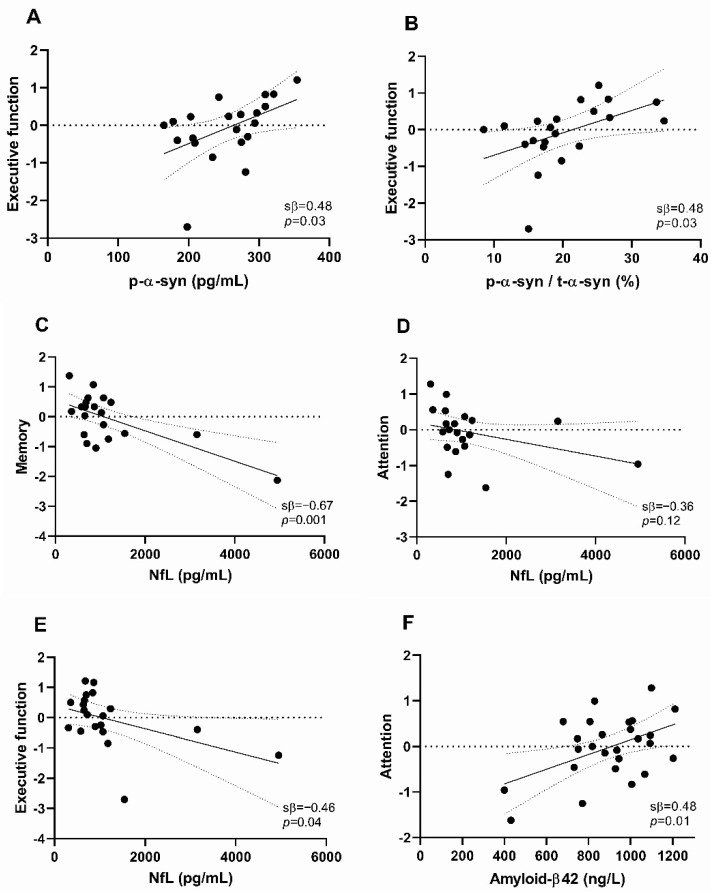

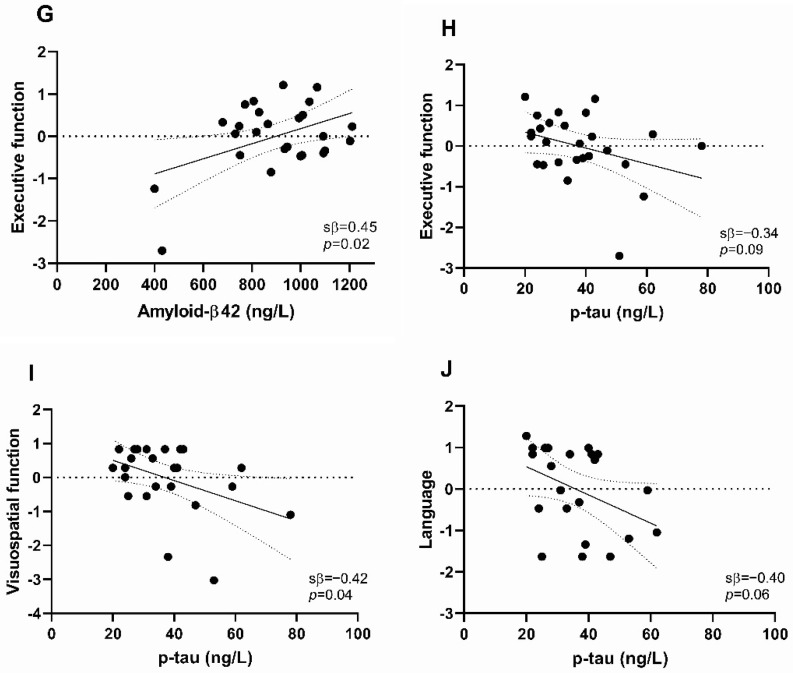
Scatter plots illustrating the relatively strongest associations of cerebrospinal fluid (CSF) biomarker levels with cognitive domain scores in early-stage Parkinson′s disease patients. Associations between (**A**) CSF p-α-syn and executive functioning, (**B**) CSF p-α-syn/t-α-syn ratio and executive functioning, (**C**) CSF NfL and memory, (**D**) CSF NfL and attention, (**E**) CSF NfL and executive function, (**F**) CSF amyloid-β42 and attention, (**G**) CSF amyloid-β42 and executive function, (**H**) CSF p-tau and executive function, (**I**) CSF p-tau and visuospatial function, (**J**) CSF p-tau and language. Data are standardized regression coefficients from univariable linear regression models. A solid line indicates a linear regression, while dotted lines have 95% confidence intervals. Each dot in the scatter plot represents a subject. Abbreviations: CSF, cerebrospinal fluid. p-α-syn, phosphorylated α-synuclein. t-α-syn, total α-syn. NfL, neurofilament light chain. p-tau, phosphorylated tau. sβ, standardized β-coefficient.

To assess relationships between CSF biomarker levels and performance on the five cognitive domains, we performed univariable and multivariable regression analyses. Associations of CSF biomarker levels with cognitive domain scores are presented in Figure 1 and Figure 2 as well as Supplementary Table S1. Associations of CSF biomarker levels with neuropsychological test scores are shown in Supplementary Figure S1.

We found no significant association of single CSF t-α-syn levels with cognitive functioning in univariable and multivariable models (Figure 1, Table S1). CSF p-α-syn was positively associated with executive functioning in both models (univariable: sβ = 0.48, *p* = 0.03, multivariable: sβ = 0.41, *p* = 0.01) (Figure 1 and Figure 2A, Table S1). Similarly, the CSF p-α-syn/t-α-syn ratio was positively associated with executive functioning (sβ = 0.48, *p* = 0.03), also in the model in which we adjusted for age, gender, and level of education (sβ = 0.40, *p* = 0.02) (Figure 1 and Figure 2B, Table S1). Individual CSF t-α-syn levels, CSF p-α-syn levels, and CSF p-α-syn/t-α-syn ratios are presented in Supplementary Table S3.

Higher CSF NfL was associated with worse functioning in multiple cognitive domains. CSF NfL was most strongly negatively associated with memory function in both univariable and multivariable models (sβ = −0.67, *p* = 0.001, and sβ = −0.59, *p* = 0.002, respectively) (Figure 1 and Figure 2C, Table S1). In addition, in both models, we found a negative association of CSF NfL with attentional functioning (univariable: sβ = −0.36, *p* = 0.12, multivariable: sβ = −0.32, *p* = 0.12) and executive functioning (univariable: sβ = −0.46, *p* = 0.04, multivariable: sβ = −0.35, *p* = 0.06) (Figure 1 and Figure 2D,E, Table S1).

CSF Aβ42 was positively associated with attentional functioning in both models (univariable: sβ = 0.48, *p* = 0.01, multivariable: sβ = 0.35, *p* = 0.04) (Figure 1 and Figure 2F, Table S1). The association of CSF Aβ42 with executive functioning was strongly attenuated after correction for age, gender, and level of education (univariable: sβ = 0.45, *p* = 0.02, multivariable: sβ = 0.20, *p* = 0.25) (Figure 1 and Figure 2G, Table S1).

Finally, CSF t-tau was not associated with performance in any of the cognitive domains (Figure 1, Table S1). CSF p-tau was negatively associated with executive functioning, visuospatial functioning, and language in univariate models (sβ = −0.34, *p* = 0.09, sβ = −0.42, *p* = 0.04, and sβ = −0.40, *p* = 0.06, respectively) (Figure 2H–J, Table S1). After including the covariates age, gender, and level of education to the model, we found a negative association between CSF p-tau and language (sβ = −0.33, *p* = 0.12). However, the associations between CSF p-tau and executive and visuospatial functioning were strongly attenuated (sβ = −0.26, *p* = 0.13, and sβ = −0.24, *p* = 0.20, respectively) (Figure 1, Table S1).

## 3. Discussion

In this cross-sectional study, we aimed to explore whether CSF biomarkers reflecting protein pathology and axonal degeneration are associated with cognitive performance in early-stage PD patients. Particularly, higher CSF NfL was strongly associated with worse performance on memory, and also associated with worse attentional and executive functioning. Furthermore, we found a moderate association between CSF p-α-syn/t-α-syn and executive functioning, between CSF Aβ42 and attentional functioning and between CSF p-tau and language.

In this study of early-stage PD patients, attention and memory were the most commonly impaired cognitive domains. Worse performance on memory tasks may be secondary to attentional deficits, even though a previous study in PD patients showed that memory remained impaired after controlling for the effect of impaired attention [18]. Taken together with the results of previous studies, the present data indicate that an impairment of memory functions may not be restricted to the advanced stages of PD or to PD dementia, but represents an important early aspect of the cognitive profile in early-stage PD [7,8,9]. Cognitive performance of these early-stage PD patients was relatively good since only 11.5% of patients were classified as having PD-MCI compared to more than 25% in other incident or early-stage PD cohorts [2]. Our early-stage PD group performed relatively well on executive functioning (impairment in 3.8% of patients). This is in contrast to the results of two previous studies in which executive function was among the most commonly affected domains in early-stage PD (impairment in 41–100% of patients) [5,9]. Methodological differences, such as the utilized neuropsychological tests and criteria of impairment of cognitive functioning, or different disease stages may explain the inconsistencies in the frequency of affected domains among our and previous early-stage PD cohorts [5,6,9].

In particular, domains that predominantly affected our early-stage PD group were associated with CSF NfL levels. Our analyses revealed an inverse association of CSF NfL levels with performance in the memory domain, and, to a lesser extent, with attentional and executive functioning. Since CSF NfL is considered a marker of axonal injury, our results suggest that axonal degeneration may lead to loss of connectivity and play an important role in impairment in multiple cognitive domains in early stage PD. To the best of our knowledge, there have not been any previous cross-sectional studies investigating CSF NfL in relation to cognitive functioning in early-stage PD patients. However, associations of CSF NfL with impairments in various cognitive domains have been reported in Alzheimer′s and frontotemporal dementia, and multiple sclerosis [19,20].

Although test scores in the executive functioning domain were generally high with little variability, we did find an association of higher CSF p-α-syn/t-α-syn levels with better executive functioning in our patients. Since we observed a very weak association between single CSF t-α-syn levels and executive functioning and a stronger association of CSF p-α-syn levels with executive functioning, the association between the CSF p-α-syn/t-α-syn ratio and executive functioning is mostly due to CSF p-α-syn levels. We previously observed higher CSF p-α-syn/t-α-syn levels in early-stage PD patients compared to healthy controls [14], suggesting that higher CSF p-α-syn/t-α-syn levels reflect a more widespread disease process in PD. This is in contrast to our present finding of a *positive* association between CSF p-α-syn/t-α-syn and executive functioning. Additional cross-sectional and longitudinal studies including patients with a wider range of executive performance are needed to validate the potential association of CSF p-α-syn/t-α-syn levels with executive functioning.

This study showed an association between lower CSF Aβ42 and worse performance on attentional functioning. A similar relation between CSF Aβ42 levels and attentional functioning has been reported previously in a cohort of PD patients with a longer disease duration compared to our cohort [21]. Since reduced CSF Aβ42 may reflect amyloid plaque pathology, our results indicate that, even in the early stages of PD, amyloid pathology may contribute to cognitive impairment. Contrary to previous early-stage PD cohorts, we did not find an association between CSF Aβ42 and memory [7,11,12]. A larger cohort size [7,12], other neuropsychological tests used [7,11,12], or worse disease severity [7] may explain the different results in other cohorts.

Although none of the patients had language difficulties, we found a weak inverse association between CSF p-tau and language. The language domain was assessed in only two previous early-stage PD studies, but no significant association with p-tau was found [7,13]. This difference might be due to other neuropsychological tests used [7,13] or worse disease severity [7]. In another previous cohort of non-demented and demented PD patients with a mean disease duration of 10 years, CSF p-tau levels were significantly higher in patients with impaired naming compared to patients with normal language function [22]. Since CSF p-tau is considered to reflect tau pathology, our results suggest that, even in early stages, underlying tau pathology might contribute to cognitive dysfunction. A strength of our study is the fact that the early-stage PD patients were well-characterized. The comprehensive neuropsychological test battery enabled us to identify impairments on various cognitive domains. By exploring CSF biomarkers reflecting different pathological mechanisms of cognitive dysfunction, i.e., axonal degeneration, α-syn accumulation, and amyloid plaque pathology, we were able to identify promising markers of cognitive functioning in early-stage PD. However, the impact of our findings is limited by the small sample size. Nearly all associations between CSF biomarker levels and cognitive functioning were attenuated after correction for confounders. Although patients were clinically diagnosed as PD, PD diagnoses were not pathologically confirmed. One PD patient in our cohort had a very high CSF NfL level. However, we have not noticed any analytical errors or clinical explanations that could explain the high value. Furthermore, we did not account for other factors that can influence cognitive performance, such as anxiety and depression. Nevertheless, our results suggest that CSF p-α-syn/t-α-syn, CSF Aβ42, and, in particular, CSF NfL are promising markers of cognitive functioning in early-stage PD. Further validation of our findings is needed in larger early-stage PD cohorts with correction for multiple testing.

## 4. Materials and Methods

### 4.1. Study Population

Data were selected from a cross-sectional cohort of PD patients recruited between 2008 and 2010 at the movement disorders outpatient clinic of Amsterdam UMC, location VUmc, previously described in detail by Van Dijk et al. [23]. All patients were diagnosed with PD by movement disorder specialists according to the United Kingdom Parkinson′s Disease Society Brain Bank clinical diagnostic criteria [24]. The severity of the motor symptoms and disease stage were assessed using the UPDRS-III and the modified Hoehn & Yahr classification, respectively. MMSE was used to assess global cognitive function. The level of education was classified using the system of Verhage, ranging from level 1 to 7 (elementary school not finished to university) [25]. PD patients were selected if the disease duration was ≤5 years, HY stage was <3, and if both neuropsychological data and CSF samples were available. Disease duration was defined as the time between the first subjective classical motor symptoms and CSF collection. The study was approved by the local medical ethical committee of VU University Medical Center, Amsterdam. All patients gave written informed consent at study entry for the use of clinical information and CSF material for scientific research purposes.

### 4.2. Neuropsychological Assessment

Comprehensive neuropsychological testing was performed by a board-certified neuropsychologist. The testing battery covered five cognitive domains: memory functioning, attention, executive functioning, visuospatial functioning, and language. Aspects of memory were tested using the modified version of the Rey Auditory Verbal Learning Test (RAVLT) to assess delayed recall [2], and the Visual Association Test (VAT) to assess associate learning ability [26]. To measure attention, the forward condition of the Digit Span was used [2]. The Trail Making Test (TMT) part A was performed to assess visual attention [2]. Executive functioning was assessed using the Stroop Color Word Test to measure interference susceptibility (to correct for speed, we divided scores for part III by scores for part II) [2]. Executive functioning was additionally tested using a test of category fluency (production of as many animals as possible within 60 s) [2]. Rey Complex Figure immediate drawing was performed to measure visuospatial orientation and visuo-construction [27]. Language, especially word retrieval, was assessed with the short version A of the Boston Naming Test [2]. A detailed description and outcome measures of all tests are presented in Supplementary Table S2.

Patients were classified as PD-MCI if performance on at least two neuropsychological test outcomes in one or multiple cognitive domains was impaired, according to the Movement Disorder Society (MDS) level II criteria [2]. Since this study was designed prior to the publication of the MDS criteria for PD-MCI, it does not include two neuropsychological tests for the domains visuospatial function and language. Raw test outcomes were transformed to standardized scores using data of healthy controls, matched by age, gender, and level of education. Impaired performance was defined as a test outcome greater than 1.5 SD below normative means. While the MDS guidelines suggest to use a range of 1–2 SD below means for impairment [2], the rigid cut-off of 1.5 SD was used in line with previous recommendations [28] to reduce false positives. Impairment on a single cognitive domain was defined as impairment of at least one test outcome within that domain. PD dementia was diagnosed if test scores were >2 SD below normative means in at least two domains.

In order to generate composite domain scores, raw neuropsychological test scores not corrected for age, gender, and level of education, were standardized to z-scores. TMT and Stroop test scores were linearly converted so that lower scores reflect worse performance. Five composite domain scores were formed by calculating mean z-scores from different test outcomes within the same domain. These domain z-scores were used to investigate associations with CSF biomarkers.

### 4.3. CSF Biomarkers

CSF was obtained by lumbar puncture between the 3rd and 4th or the 4th and 5th lumbar vertebrae, as previously described [22]. CSF was routinely assayed for cell count, centrifuged at 1800× *g* for 10 min at room temperature and, thereafter, aliquoted in 0.5 mL polypropylene tubes, and stored at −80 °C [29]. Concentrations of CSF t-α-syn, p-α-syn, NfL, Aβ42, t-tau, and p-tau were measured using enzyme-linked immunosorbent assays (ELISAs), as published elsewhere [14,23,30].

### 4.4. Statistical Analysis

For statistical analyses, SPSS 24 for Windows (SPSS Inc., Chicago, IL, USA) was used. We first determined the frequency of PD-MCI and PD dementia, and the frequency of impairment across different cognitive domains. Then, to explore the relationship between CSF biomarkers and cognitive functioning, a linear regression analysis was used with the composite domain scores as dependent variables and CSF biomarker levels as independent variables. For each variable, first univariable linear regression analyses were performed. Then, multivariable linear regression analyses including the potential confounders age, gender, and level of education were used [31].

## 5. Conclusions

In conclusion, in our cohort of early-stage PD patients, attention and memory are among the most frequently affected cognitive domains. Our results show that CSF p-α-syn/t-α-syn, CSF Aβ42, p-tau, and, in particular, CSF NfL levels are associated with cognitive performance in early-stage PD patients. This indicates that CSF biomarkers reflecting protein pathology and axonal degeneration may identify early-stage PD patients who are at an increased risk of developing cognitive impairment.

## Figures and Tables

**Table 1:** Demographic data and cerebrospinal fluid concentrations in patients with early-stage Parkinson′s disease.

Demographics	*n*	
Female/male (*n*)	26	14/12
Age (years)	26	63 ± 13 (41–84)
Education (number per Verhage level 1/2/3/4/5/6/7)	26	0/1/1/3/8/5/8
Disease duration (years)	26	2 (1–5)
Hoehn and Yahr stage (nr. per stage 1/1.5/2/2.5/3/4/5)	26	2/3/13/8/0/0/0
UPDRS III score	26	22 (6–38)
MMSE score	26	29 (23–30)
BDI score	22	8 (0–29)
BAI score	22	10 (1–21)
**CSF Biomarker Levels**		
t-α-syn (pg/mL)	21	1271 (723–1950)
p-α-syn (pg/mL)	21	268 (165–354)
p-α-syn/t-α-syn (%)	21	18.9 (8.5–34.7)
NfL (pg/mL)	20	858 (307–4948)
Amyloid-β_42_ (ng/L)	26	931 (400−1211)
t-tau (ng/L)	26	186.5 (95.0–369.0)
p-tau (ng/L)	26	35.5 (20.0–78.0)

Data are mean ± SD and range or median and range unless specified otherwise. Abbreviations: CSF, cerebrospinal fluid. UPDRS-III, Unified Parkinson′s Disease Rating Scale Part III. MMSE, Mini-Mental State Examination. t-α-syn, total α-synuclein. p-α-syn, phosphorylated α-synuclein. t-tau, total tau. p-tau, phosphorylated tau. NfL, neurofilament light chain. BDI Beck Depression Inventory. BAI, Beck Anxiety Inventory.

**Table 2:** Neuropsychological test scores and cognitive domain scores.

Cognitive Domain	Test	*n*	Test Score	*t*-score ^a^
**Memory**	RAVLT delayed recall (nr. of words correct)	26	7.8 ± 3.4 (2–15)	43.3 ± 11.6 (24–66)
	VAT A trial 1 (nr. of objects correct)	26	5.6 ± 0.6 (4–6)	43.7 ± 2.3 (37–45)
	Domain z-score ^b^	26	0.00 ± 0.80 (−2.13–1.37)	NA
**Attention**	TMT A (sec)	26	45 ± 20 (19–116)	43.6 ± 8.9 (26–58)
	Digit Span forward (nr. of series correct)	25	13 ± 3 (6–19)	49.8 ± 17.1 (16–75)
	Domain z-score ^b^	26	−0.02 ± 0.72 (−2.32–1.08)	NA
**Executive functions**	Category fluency (nr. of words correct)	26	24 ± 5 (14–32)	50.7 ± 8.8 (35–69)
	Stroop Color Word (sec part III / sec part II)	24	1.8 ± 0.4 (1.2–3.2)	50.0 ± 8.2 (32–67)
	Domain z-score ^b^	26	0.00 ± 0.81 (−2.70–1.21)	NA
**Visuospatial function**	Rey copy (elements correct)	25	33 ± 3.6 (22–36)	38.6 ± 3.4 (28–40)
	Domain z-score ^c^	25	0.00 ± 1.00 (−3.03–0.83)	NA
**Language**	Boston Naming Test (nr. of objects correct)	23	78 ± 7 (67–87)	47.5 ± 7.8 (36–65)
	Domain z-score ^c^	23	0.00 ±1.00 (−1.63–1.28)	NA

Data are mean ± SD and range.^a^ neuropsychological test scores transformed to external normative test scores, adjusted for age, gender, and level of education.^b^ domain z-scores are mean z-scores from two tests of the same domain.^c^ domain z-scores are z-scores from Rey copy or Boston Naming Test, respectively. Abbreviations: RAVLT, Rey Auditory Verbal Learning Test. VAT, Visual Association Test. TMT, Trail Making Test.

## Supplementary Materials

Supplementary materials can be found at https://www.mdpi.com/1422-0067/21/22/8519/s1. Table S1. Results of the Linear Regression Analyses Relating CSF Biomarker Levels to Cognitive Domain Scores. Table S2. Detailed Description of Neuropsychological Tests and Outcome Measures., Table S3. Individual Levels of CSF Total α−synuclein and Phosphorylated α−synuclein in 21 Patients with Early−stage Parkinson’s Disease. Figure S1: Associations of CSF biomarker levels with neuropsychological test scores.

## Abbreviations

Aβ42	amyloid-β_42_
AD	Alzheimer′s disease
CSF	cerebrospinal fluid
ELISA	enzyme-linked immunosorbent assays
HY	modified Hoehn & Yahr classification
NfL	neurofilament light chain
MDS	Movement Disorder Society
MMSE	Mini-Mental State Examination
p-α-syn	α-synuclein phosphorylated at residue Ser 129
PD	Parkinson′s disease
PD-MCI	Parkinson′s disease mild cognitive impairment
p-tau	tau phosphorylated at threonine 181
RAVLT	Rey Auditory Verbal Learning Test
t-α-syn	total α-synuclein
t-tau	total tau
TMT	Trail Making Test
UPDRS III	Unified Parkinson′s Disease Rating Scale Part III
VAT	Visual Association Test
