# Supplementary material for: CSF Biomarkers Reflecting Protein Pathology and Axonal Degeneration Are Associated with Memory, Attentional, and Executive Functioning in Early-Stage Parkinson′s Disease
Source: Int J Mol Sci. 2020 Nov 12;21(22):8519. doi: 10.3390/ijms21228519 (PMC7697681; doi:10.3390/ijms21228519)
Supplement: Supplementary file 1 [file ijms-21-08519-s001.pdf]

## SUPPLEMENTARY DATA

**Table S1.** Results of the Linear Regression Analyses Relating CSF Biomarker Levels to Cognitive Domain Scores.

| | | Univariable Linear Regression | | Multivariable Linear Regression* | |
|---|---|---|---|---|---|
| **Independent Variable** | **Dependent Variable** | **sβ (95% CI)** | ***p*** | **sβ (95% CI)** | ***p*** |
| CSF t−α−syn (pg/mL) | Memory | −0.277 (−0.810–0.201) | 0.223 | −0.154 (−0.672–0.335) | 0.488 |
| | Attention | −0.085 (−0.561–0.392) | 0.715 | 0.143 (−0.278–0.562) | 0.484 |
| | Executive function | −0.189 (−0.687–0.293) | 0.411 | −0.079 (−0.497–0.333) | 0.680 |
| | Visuospatial function | −0.360 (−0.864–0.107) | 0.119 | −0.146 (−0.652–0.344) | 0.520 |
| | Language | −0.325 (−0.864–0.169) | 0.174 | −0.198 (−0.787–0.364) | 0.443 |
| CSF p−α−syn (pg/mL) | Memory | 0.061 (−0.557–0.720) | 0.793 | −0.018 (−0.608–0.559) | 0.930 |
| | Attention | −0.043 (−0.521–0.435) | 0.853 | −0.134 (−0.528–0.261) | 0.483 |
| | Executive function | 0.479 (0.060–0.936) | 0.028 | 0.411 (0.108–0.747) | 0.012 |
| | Visuospatial function | −0.035 (−0.574–0.499) | 0.885 | −0.095 (−0.583–0.376) | 0.652 |
| | Language | −0.143 (−0.690–0.386) | 0.559 | −0.182 (−0.735–0.347) | 0.455 |
| CSF p−α−syn/t−α−syn | Memory | 0.141 (−0.367–0.676) | 0.542 | −0.005 (−0.516–0.506) | 0.983 |
| | Attention | −0.044 (−0.521–0.434) | 0.851 | −0.259 (−0.662–0.146) | 0.195 |
| | Executive function | 0.479 (0.060–0.937) | 0.028 | 0.402 (0.065–0.771) | 0.023 |
| | Visuospatial function | 0.249 (−0.247–0.779) | 0.291 | 0.069 (−0.428–0.577) | 0.757 |
| | Language | 0.154 (−0.370–0.695) | 0.529 | 0.012 (−0.561–0.586) | 0.963 |
| CSF NfL (pg/mL) | Memory | −0.667 (−1.059–0.308) | 0.001 | −0.588 (−0.938–0.268) | 0.002 |
| | Attention | −0.357 (−0.852–0.110) | 0.122 | −0.316 (−0.741–0.094) | 0.119 |
| | Executive function | −0.461 (−0.992–0.026) | 0.041 | −0.347 (−0.780–0.013) | 0.059 |
| | Visuospatial function | −0.136 (−0.670–0.389) | 0.578 | −0.024 (−0.529–0.475) | 0.916 |
| | Language | 0.004 (−0.497–0.507) | 0.986 | 0.160 (−0 324–0.637) | 0.491 |
| CSF Aβ42 (ng/mL) | Memory | 0.321 (−0.078–0.721) | 0.110 | 0.110 (−0.293–0.513) | 0.577 |
| | Attention | 0.483 (0.113–0.851) | 0.012 | 0.354 (0.012–0.697) | 0.043 |
| | Executive function | 0.446 (0.070–0.823) | 0.022 | 0.200 (−0.155–0.554) | 0.253 |
| | Visuospatial function | 0.123 (−0.338–0.611) | 0.558 | 0.079 (−0.342–0.518) | 0.674 |
| | Language | −0.014 (−0.510–0.480) | 0.950 | −0.049 (−0.543–0.435) | 0.820 |
| CSF t−tau (ng/L) | Memory | −0.211 (−0.623–0.200) | 0.301 | −0.128 (−0.526–0.269) | 0.511 |
| | Attention | −0.055 (−0.476–0.366) | 0.790 | 0.145 (−0.223–0.513) | 0.422 |
| | Executive function | −0.179 (−0.593–0.236) | 0.382 | −0.119 (−0.476–0.238) | 0.496 |
| | Visuospatial function | −0.404 (−0.786–0.009) | 0.045 | −0.179 (−0.575–0.223) | 0.368 |
| | Language | −0.312 (−0.807–0.129) | 0.147 | −0.184 (−0.695–0.296) | 0.409 |
| CSF p−tau (ng/L) | Memory | −0.270 (−0.676–0.135) | 0.181 | −0.156 (−0.548–0.235) | 0.415 |
| | Attention | −0.163 (−0.579–0.253) | 0.426 | 0.052 (−0.317–0.421) | 0.773 |
| | Executive function | −0.342 (−0.738–0.054) | 0.087 | −0.255 (−0.593–0.083) | 0.132 |
| | Visuospatial function | −0.424 (−0.813–0.033) | 0.035 | −0.243 (−0.626–0.140) | 0.201 |
| | Language | −0.396 (−0.993–0.026) | 0.062 | −0.329 (−0.916–0.113) | 0.119 |

sβ =standardized regression coefficient * including the variables age, gender, and level of education. Abbreviations: CSF, cerebrospinal fluid; t−α−syn, total α−synuclein; p−α−syn, phosphorylated α−synuclein; NfL, neurofilament light chain; Aβ42, amyloid−β42; t−tau, total tau; p−tau, phosphorylated tau.

**Table S2.** Detailed Description of Neuropsychological Tests and Outcome Measures

| Neuropsychological Test | Test Variable Used | Cognitive Component | Description | Outcome Measure |
|---|---|---|---|---|
| **Memory** | | | | |
| Rey Auditory Verbal Learning Test (RAVLT) | Delayed recall | Free recall | A list of 15 unrelated words are read to the subjects, repeated over 5 trials. After a delay of 20 min, subjects are asked to recall as many words as possible. | Number of words Range: 0–15 |
| Visual Attention Test (VAT) | Part A trial 1 | Automatic learning | Subjects are asked to recall unusual pairs of objects (e.g. a monkey with an umbrella). | Number of objects Range: 0–6 |
| **Attention** | | | | |
| Trail Making Test | Part A | Attention | Subjects are asked to connect ascending numbers as quickly as possible. | Time needed to complete (seconds) |
| WAIS–III Digit Span | Forward condition | Attention | Subjects are asked to recall series of numbers in a correct order. | Number of correct series Range: 0–21 |
| **Executive function** | | | | |
| Stroop Color Word interference test | Card 3/2 | Mental set shifting/ response inhibition | Subjects need to name series of colors and words, which reflects processing speed (card 1 and 2). On card 3, names of words are printed in conflicting colors (e.g. red is printed in blue) and subjects need to name the color of the ink instead of the word. | Time needed on card 3 / time needed on card 2 (seconds) |
| Category Fluency | – | Working memory/ mental set shifting | Subjects are asked to produce as many animals as possible within a time span of 60 s. | Number of animals |
| **Visuospatial function** | | | | |
| Rey Complex Figure | Copy | Visuospatial orientation Visuoconstruction | Subjects are asked to copy a complex figure. The figure consists of 18 elements. | Score based on drawn elements Range: 0–36 |
| **Language** | | | | |
| Boston Naming Test–short version A | – | Word retrieval | Subject are asked to name objects from 29 line drawings. | Score based on correctly named objects Range: 0–87 |

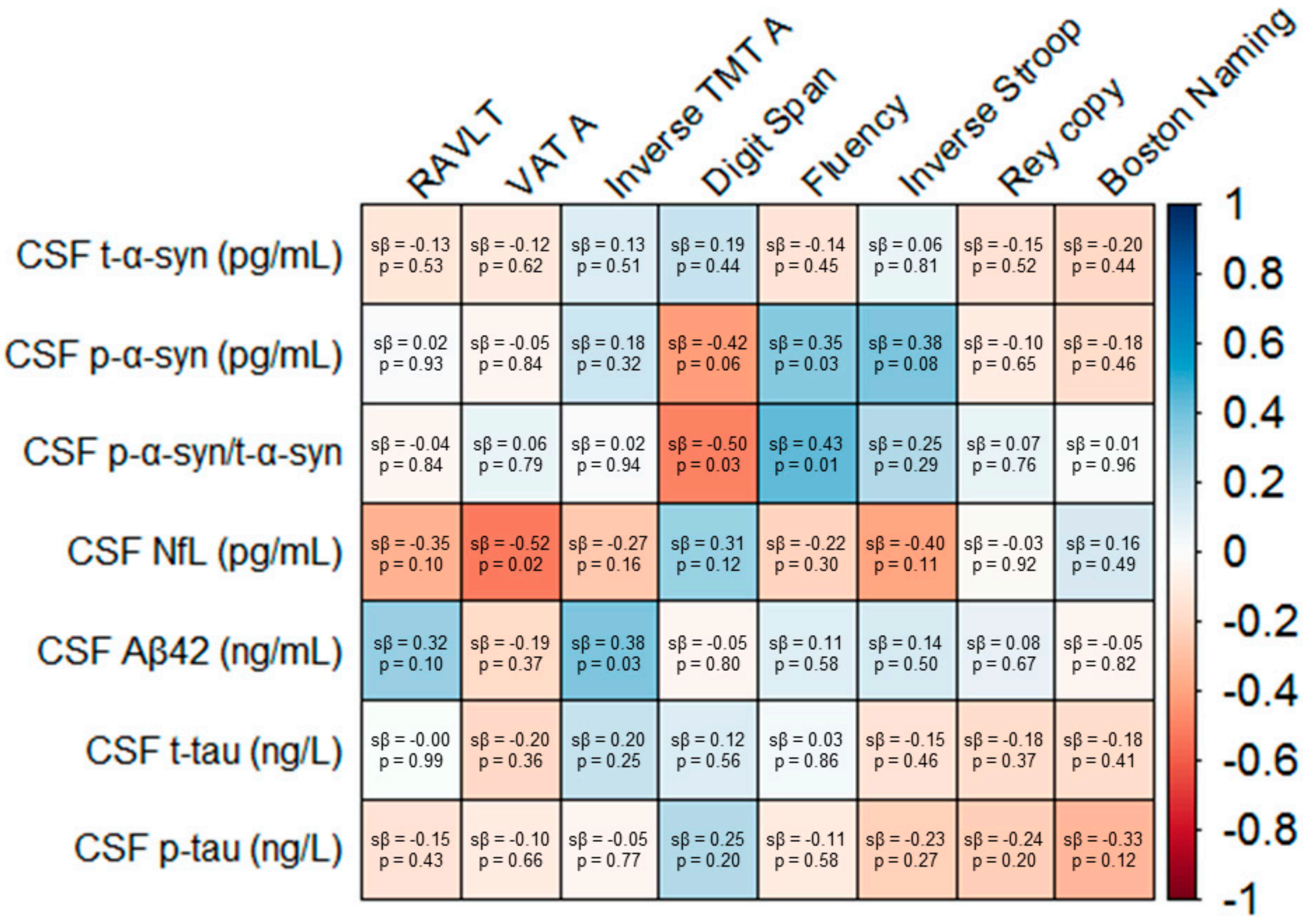


**Figure S1.** Associations of CSF biomarker levels with neuropsychological test scores. Data are standardized β coefficients from linear regression models, adjusted for age, gender, and level of education. Color intensity is based on the standardized β coefficients. Abbreviations: CSF, cerebrospinal fluid; t−α−syn, total α−synuclein; p−α−syn, phosphorylated α−synuclein; NfL, neurofilament light chain; Aβ42, amyloid−β42; t−tau, total tau; p−tau, phosphorylated tau; RAVLT, Rey Auditory Verbal Learning Test; VAT, Visual Association Test; TMT, Trail Making Test.

**Table S3.** Individual Levels of CSF Total $\alpha$−synuclein and Phosphorylated $\alpha$−synuclein in 21 Patients with Early−stage Parkinson's Disease

| Subject | CSF t−$\alpha$−syn (pg/mL) | CSF p−$\alpha$−syn (pg/mL) | CSF p−$\alpha$−syn/t−$\alpha$−syn (%) |
|---|---|---|---|
| 1 | 1436 | 274 | 19,1 |
| 2 | 723 | 243 | 33,6 |
| 3 | 1111 | 297 | 26,8 |
| 4 | 1323 | 198 | 15 |
| 5 | 1186 | 234 | 19,8 |
| 6 | 740 | 257 | 34,7 |
| 7 | 1551 | 178 | 11,5 |
| 8 | 1802 | 284 | 15,7 |
| 9 | 1416 | 268 | 18,9 |
| 10 | 1247 | 203 | 16,3 |
| 11 | 1401 | 354 | 25,2 |
| 12 | 1613 | 294 | 18,2 |
| 13 | 1217 | 209 | 17,2 |
| 14 | 1187 | 206 | 17,4 |
| 15 | 1225 | 275 | 22,4 |
| 16 | 1271 | 184 | 14,5 |
| 17 | 1950 | 165 | 8,5 |
| 18 | 1710 | 281 | 16,4 |
| 19 | 1263 | 309 | 24,5 |
| 20 | 1371 | 309 | 22,6 |
| 21 | 1205 | 321 | 26,6 |

Abbreviations: CSF, cerebrospinal fluid; t−$\alpha$−syn, total $\alpha$−synuclein; p−$\alpha$−syn, phosphorylated $\alpha$−synuclein.
